# 3D-Printed Multifunctional Multicompartment Polymer-Based Capsules for Tunable and Spatially Controlled Drug Release

**DOI:** 10.3390/jfb16120456

**Published:** 2025-12-08

**Authors:** Antonio Minopoli, Giordano Perini, Davide Evangelista, Matteo Marras, Alberto Augello, Valentina Palmieri, Marco De Spirito, Massimiliano Papi

**Affiliations:** 1 Dipartimento di Neuroscienze, Università Cattolica del Sacro Cuore, Largo Francesco Vito 1, 00168 Rome, Italy; antonio.minopoli@unicatt.it (A.M.); giordano.perini@unicatt.it (G.P.); davide.evangelista@guest.policlinicogemelli.it (D.E.); matteo.marras@guest.policlinicogemelli.it (M.M.); marco.despirito@unicatt.it (M.D.S.); 2 Fondazione Policlinico Universitario A. Gemelli IRCSS, 00168 Rome, Italy; alberto.augello@policlinicogemelli.it; 3 Istituto dei Sistemi Complessi, Consiglio Nazionale delle Ricerche, CNR, via dei Taurini 19, 00185 Rome, Italy; valentina.palmieri@cnr.it

**Keywords:** 3D printing, biopolymer-based capsules, controlled drug release, edible hydrogels, enteric coating, essential oil, oil microemulsion

## Abstract

The development of polymer-based systems is central to the design of next-generation drug delivery carriers, as polymers enable versatile tuning of physicochemical properties and responsiveness. In this work, we introduce a 3D printing-based strategy for the fabrication of multicompartment capsules that integrate multiple polymers within a unique one-step process. This approach allows precise spatial organization and structural complexity, yielding capsules with customizable features such as compartmentalization, polymer-specific responsiveness, and localized release control. In particular, pH-triggered release can be programmed across distinct polymeric regions of the capsules, enabling site-specific delivery along different intestinal segments, including the small intestine and colon. The use of 3D printing thus provides a scalable and adaptable platform to generate multifunctional polymer-based carriers with finely tunable drug release profiles, paving the way for new directions in polymer-enabled controlled delivery technologies.

## 1. Introduction

3D-printed capsules for enteric drug delivery represent an emerging technology with significant potential to transform oral therapeutics by enabling patient-specific dosing, precise control of release kinetics, and the creation of complex, multicompartmental structures that enhance targeting within the gastrointestinal tract [1,2,3]. Through additive manufacturing, these systems can integrate enteric coatings or compartmentalized drug loading to protect active ingredients from gastric acidity and ensure site-specific intestinal release [4,5]. Despite these advantages, clinical and commercial translation is hindered by critical challenges, including the limited availability of pharmaceutical-grade printable polymers with proven biocompatibility and mechanical strength, the risk of structural fragility in low-density designs, slower production rates compared to conventional capsule manufacturing, and regulatory frameworks that are not yet adapted to additive manufacturing [3,6,7].

Recent studies have reported several strategies to overcome these limitations [8,9,10,11]. Extrusion-based printing has enabled the fabrication of single- or dual-layer capsules with enteric protection, but such approaches often lack the ability to finely modulate release profiles beyond a binary “on–off” behavior [12,13,14]. Other works have exploited polymer blends or functional coatings to impart responsiveness to pH, temperature, or enzymatic activity; however, these methods typically require sequential processing steps and are less effective at generating complex architectures with distinct functional domains. Moreover, post-processing or coating stages introduce variability and increase the risk of inter-batch inconsistency, which remains a barrier to reproducibility and scalability [15,16,17,18,19,20].

In contrast, our approach introduces a fully integrated 3D-printed process in which compartment filling and sealing occur in one continuous automated workflow. Each capsule can be produced in less than 2 min (excluding the Eudragit drying phase), with a 98% reproducibility rate for shell fabrication and >90% reproducibility for inner-compartment formation. The platform is inherently scalable, and production time per capsule is expected to decrease further in high-throughput settings, where the dead time associated with cartridge changes is minimized. By combining different polymers in spatially organized compartments, this approach allows for the direct integration of polymer-specific responsiveness within the capsule structure, eliminating the need for additional coating or processing stages. Compared with conventional extrusion or coating-based systems, our method offers several advantages: (i) enhanced structural complexity through programmable spatial patterning, (ii) tailored release kinetics enabled by compartmentalized polymer domains and polymer crosslinking, and (iii) improved design versatility that can be adapted to specific therapeutic needs, including site-targeted release along different intestinal segments. Importantly, the multicompartmental design not only enables multi-drug loading with independent release triggers but also allows the physical separation of compounds that would otherwise be incompatible in a single formulation—for instance, preventing direct contact between probiotics and lipids (e.g., essential oils), thereby preserving their stability and efficacy [21,22,23].

**Figure 1:**
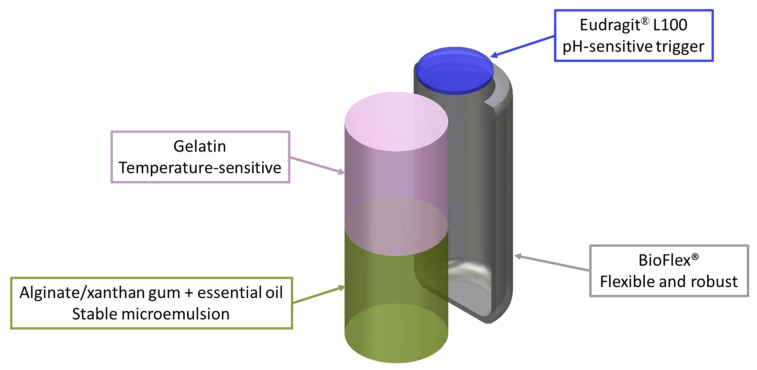
Sketch of the printed capsule consisting of two distinct compartments:The innermost compartment, in green, houses a microemulsion made of alginate, xanthan gum, and rosemary essential oil, whereas the outermost compartment, in pink, is a gelatin-based blend. The capsule shell, in grey, is made with BioFlex^®^ while the capsule lid, in blue, is made with Eudragit^®^ L100, which offers acidic resistance at pH below 6.0.

In the system developed, each compartment is engineered with a specific polymeric material serving a defined function (Figure 1): Eudragit L100, deposited on the capsule lid, provides gastroresistance and acts as a pH-sensitive trigger for intestinal release; BioFlex polymer forms the capsule shell, combining mechanical robustness with flexibility to facilitate swallowing and promote content release under peristaltic pressure [24]; xanthan gum enables incorporation of essential oils into a stable microemulsion; alginate blended with xanthan gum creates a viscous gel matrix that mediates sustained essential oil release; and gelatin forms the upper layer potentially encapsulating probiotics and ensuring their earlier release compared to the essential oil. Notably, gelatin also displays a favorable sol–gel transition, remaining solid at room temperature but becoming more viscous under physiological conditions, thereby promoting controlled release. Beyond their functional roles, the selected materials—xanthan gum, alginate, and gelatin—are already available at an industrial scale for commercial applications and are widely recognized as edible, biocompatible, and pharmacologically inert, minimizing risks of undesired interactions with co-administered drugs.

## 2. Materials and Methods

### 2.1. Chemicals and Materials

BioFlex^®^ (FILOALFA, Turin, Italy) was used to print the capsule shell. Sodium alginate (CAS No. 9005-38-3), xanthan gum from *Xanthomonas campestris* (CAS No. 11138-66-2), gelatin from porcine skin (CAS No. 9000-70-8), triethyl citrate (CAS No. 77-93-0), phosphate-buffered saline (pH 7.4), and Rhodamine B (CAS No. 81-88-9) were purchased from Sigma-Aldrich (St. Louis, USA). Rosemary essential oil was purchased from a local shop. Eudragit^®^ L100 was purchased from Evonik Operations GmbH and was selected for its pH-dependent solubility that allows dissolution at pH ≥ 6.0, ensuring selective release in the small intestine.

### 2.2. Bioink and Polymer Preparation

Sodium alginate at 3% *w*/*v* concentration and xanthan gum at 1.5% *w*/*v* were dissolved in sterile water under vigorous stirring at 80 °C. After 2 h, rosemary essential oil was added in the ratio of 1:3 (*v*/*v*). The mixture was stirred overnight at room temperature. Rhodamine B was added to a 10% gelatin at a concentration of 175 nM; the blend was pre-warmed at 37 °C during the printing step. Eudragit L100 was dissolved in 90% ethanol in the ratio 1:10 *w*/*w* under vigorous stirring at room temperature overnight. Triethyl citrate at 15% *w*/*w* was added to the Eudragit blend as a plasticizer. Biopolymers were kept at 4 °C in dark conditions until use.

### 2.3. Capsule Biofabrication

Hollow capsules were printed using a fused deposition modeling printer (X1 Carbon, Bambu Lab, Shenzen, China) with a 0.4 mm diameter nozzle. The BioFlex filament was printed at 230 °C. Capsules were filled using an extrusion bioprinter (BIO X6, Cellink, Gothenburg, Sweden) equipped with volumetric-controlled syringes. A volume of 300 µL of oil-based bioink was dispensed through an 18-gauge blunt needle. Then, 300 µL of gelatin/rhodamine B was subsequently dispensed to fill the capsule. Eventually, 5 µL of Eudragit solution was extruded to create a gastroprotective film of thickness 0.3 mm. Capsules were kept at room temperature in dark conditions for 30 min, allowing Eudragit film to dry. 

### 2.4. Morphology and Mechanical Testing

To assess the surface morphology of the capsules, a flat BioFlex specimen (diameter 10 mm) was fabricated using the same printing parameters adopted for the capsules. This approach allowed a more accurate evaluation of the surface morphology, which could not be reliably analyzed on the curved capsule shell. Samples were soaked in distilled water at pH 2.5 for 2 h. Morphology and contact angle were evaluated through the profilometer and contact angle modules, respectively, of Attension Theta Flow (Biolin Scientific AB, Gothenburg, Sweden).

3D printed dog-bone-shaped specimens were tested with a mechanical analyzer (UniVert CellScale System, Waterloo, Canada). The grip separation was 20 mm, and the speed rate was 10 mm/s with a maximum displacement of 25 mm. At least three samples for each condition were used. Additionally, 3D printed capsules were tested in compression with a speed rate of 0.6 mm/s with a total displacement of 6 mm. Force displacement curves were extracted.

### 2.5. Fluorescence Confocal Microscopy

Size distributions of oil droplets in alginate/essential oil and alginate/xanthan gum/essential oil blends were measured, taking advantage of rosemary essential oil autofluorescence under 350–400 nm excitation wavelength. Fluorescence images were acquired with a confocal laser scanning microscope FLUOVIEW FV4000 (Evident Scientific, Tokyo, Japan) equipped with a 405-nm solid-state laser. Fluorescent oil droplets were analyzed with the “Analyze Particles” tool implemented in Fiji/ImageJ (Version 1.54p). Droplet diameters (D) were retrieved from droplet area (A) as $D=2\sqrt{A/\pi }$.

### 2.6. Release Assay, Storage Stability, and Compartmentalization Efficacy

**Figure 2:**
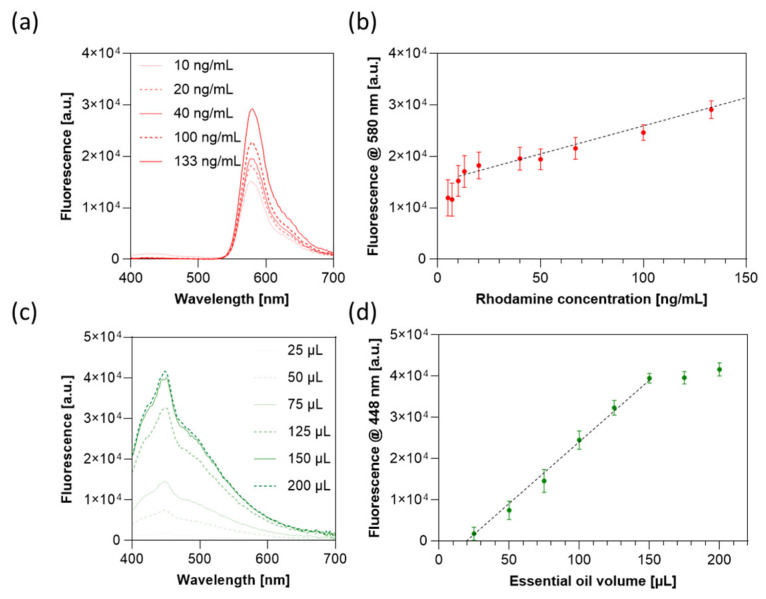
Fluorescence spectra as a function of (**a**) rhodamine B concentration and (**c**) rosemary essential oil volume. Calibration curves obtained from the emission peaks at (**b**) 580 nm for rhodamine B and (**d**) 448 nm for rosemary essential oil. Data are represented as mean value ± standard deviation of three independent experiments. Dotted lines highlight the corresponding linear range: 10–133 ng/mL for rhodamine B and 25–150 µL for rosemary essential oil.

In order to test the release profile, capsules were kept in phosphate-buffered saline (PBS) at pH 2.5 for 2 h and then transferred to pH 7.4 for 6 h under vigorous stirring (320 rpm) at 37 °C. The fluorescence spectrum of the eluate was recorded with a Cytation 3 Cell Imaging Multi-Mode Reader (BioTek, Terrebonne, Canada). The fluorescence spectra of rhodamine B and rosemary essential oil (autofluorescence) were acquired under 370 nm excitation wavelength. Emission peaks of rhodamine B and rosemary essential oil at 580 nm and 448 nm, respectively, were measured over time to retrieve the release profile. To quantify the released compounds, fluorescence calibration curves were established for both rhodamine B (Figure 2a) and rosemary essential oil (Figure 2c). A linear correlation ($R^{2}$ > 0.99) was observed between fluorescence intensity and concentration for rhodamine B within the range of 10–133 ng/mL (Figure 2b), and for rosemary essential oil volumes ranging from 25 to 150 µL (Figure 2d).

Capsule stability was assessed over a 7-day period by monitoring capsule weight under controlled temperature (4 °C and 25 °C) and humidity (50% RH) conditions. Compartmentalization efficacy was evaluated using a squared capsule model with a removable face, fabricated according to the printing protocol described in Section 2.3. Immediately after printing and after 2 h of incubation at 25 °C and 50% RH, the capsule contents were imaged via confocal microscopy to examine the interface definition and identify any cross-contamination or dye diffusion across the compartment boundary.

### 2.7. Numerical Simulation (Finite Element Method, FEM)

A time-dependent 2D model was developed in COMSOL Multiphysics 6.2 to represent a capsule compartmentalized into two sections. The capsule was modeled as two adjoining compartments, each 10 × 10 mm^2^, embedded in a water domain of 60 × 100 mm^2^. A porosity of 0.8 was assigned to the capsule infill hydrogels, incorporating two solute species, *c*_1_ and *c*_2_. Transport in porous media was captured by coupling the Transport of Diluted Species in Porous Media interface (advection–diffusion–dispersion within the porous subdomain) with Free and Porous Media Flow (Brinkman formulation in porous regions and laminar flow in the lumen). The interfaces were linked via standard Multiphysics couplings to enforce continuity of velocity and species flux across internal boundaries. Initial concentrations were set to 1 M, while the water inflow was a sinusoidal velocity function $v=\sin\left(2\pi t\right)\cdot$0.1 m/s. The capsule aperture was varied in the range 0.2–1.0 cm. The simulation spanned 2 h with a time step of 0.1 h. Release from both compartments was evaluated as the residual concentration in the corresponding compartment as a function of time.

## 3. Results

### 3.1. Numerical Simulation-Driven Capsule Design

**Figure 3:**
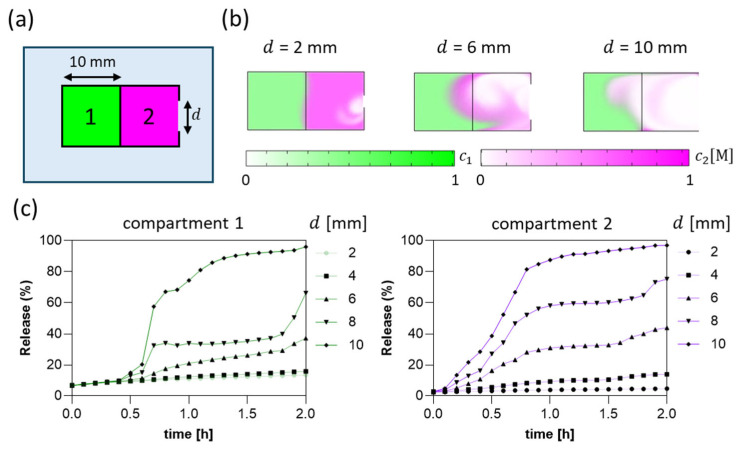
(**a**) Simulation workspace consisting of a rectangular capsule divided into two compartments housing alginate/xanthan gum/essential oil (left side, compartment 1) and gelatin (right side, compartment 2). The capsule is placed in a pipe, in which water flows horizontally with an oscillatory function. (**b**) Concentration distributions after 1 h as a function of aperture diameter. (**c**) Percentage of release from compartments 1 and 2 as a function of aperture diameter from 2 mm to 10 mm over 2 h.

To optimize the structural parameters of the capsule and achieve controlled drug release from its two compartments, we conducted a series of in silico simulations using COMSOL Multiphysics. The model was based on a 10 × 20 mm^2^ rectangular geometry with two 10 × 10 mm^2^ compartments (Figure 3a), designed to replicate the aspect ratio and dimensions of some standard oral capsules. The content of each compartment is modeled to resemble the mechanical properties of hydrogel blends, such as viscosity and porosity. The aperture of the capsule is located on the shortest side and has a variable diameter. Numerical analyses were performed to study the release kinetics as a function of the aperture diameter on the capsule surface.

The capsule is placed in a pipe, in which we set an oscillatory fluid flow at a velocity of 10 cm/min, characteristic of the intestinal environment [25] (Figure 3b), allowing us to estimate the influence of mechanical perturbations on diffusive and convective mass transport. The results shown in Figure 3c revealed that the compartment closer to the aperture released its contents efficiently, even with small openings (2 mm). In contrast, the innermost compartment exhibited incomplete release for aperture d ≤ 8 mm (~60% after 2 h) due to limited fluid penetration and restricted mass transfer through narrow apertures. This effect is safe due to the stagnation points generated by the squared geometry used in our simulation, which will not occur in the experimental setup, where a blunted geometry was adopted to facilitate swallowing. Consequently, these results guided the selection of the capsule aspect ratios (i.e., 0.8 aperture diameter over capsule diameter and 0.4 aperture diameter over capsule length) and blunted internal geometry to ensure that, upon the dissolution of the enteric coating, the physical design would support the complete release of the content.

### 3.2. Hollow Capsule Fabrication

**Figure 4:**
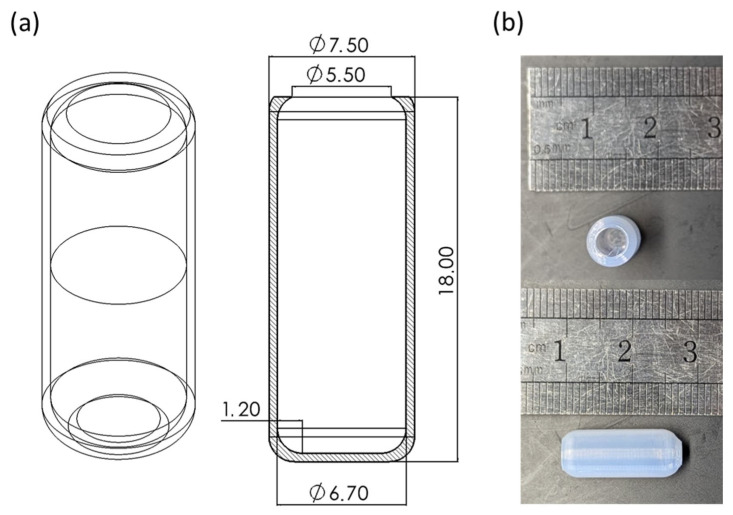
(**a**) Technical CAD rendering and (**b**) top and side views of the fabricated capsule highlighting the dimensional accuracy achieved with the 3D printing process. Dimensions in panel (**a**) are reported in millimeters.

The capsules were designed in a blunted cylindrical shape with aspect ratios inferred from numerical studies: length (l) of 18.0 mm, diameter (d) of 7.5 mm, and aperture (a) of 5.5 mm (Figure 4a) to replicate the dimensions of standard commercial enteric capsules (Size #0) and comply with Food and Drug Administration (FDA) and European Medicines Agency (EMA) guidelines for oral dosage form size [26,27]. Capsules were fabricated using a fused deposition modeling printer with BioFlex filament and a nozzle with a nominal resolution of 0.4 mm. The printed hollow capsule has dimensions of l = 17.93 ± 0.12 mm, d = 7.42 ± 0.06 mm, a = 5.44 ± 0.09 mm, and thickness (t) of 0.35 ± 0.04 mm, being consistent with the CAD model and nozzle resolution. The wall thickness was minimized to the nozzle resolution limit; this design choice was driven by the need to minimize the patient’s intake of the inert BioFlex^®^ polymer shell, reducing the non-digestible mass while preserving the structural integrity required to prevent premature leakage. The printing process demonstrated excellent reproducibility, achieving a 98% fabrication success rate across multiple samples with no visible gaps, voids, or surface defects (Figure 4b).

### 3.3. Morphological Characterization

**Figure 5:**
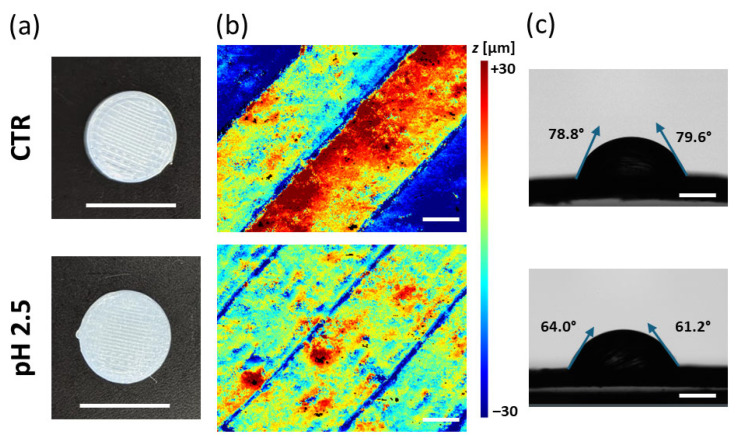
(**a**) Representative pictures of control and acid-treated BioFlex specimens used for morphological characterization. Scale bar: 10 mm. Corresponding (**b**) *z* map (scale bar: 200 µm) and (**c**) contact angle (scale bar: 500 µm).

Surface analysis revealed that the exposure of the printed capsule to an acidic environment (pH 2.5) produced a dual morphological effect. Although the overall surface roughness parameters decreased—i.e., the areal average roughness ($S_{a}$) decreased from 17.67 µm to 9.89 µm, and the quadratic mean roughness ($S_{q}$) from 21.82 µm to 13.60 µm—the vertical roughness components increased markedly (Figure 5a). In particular, the vertical total height parameter ($R_{z}$) rose from 110.85 µm to 136.02 µm, and the maximum valley depth ($R_{v}$) from 74.01 µm to 81.43 µm, suggesting that while the microscale texture became smoother due to the dissolution of minor irregularities, deeper valleys and large-scale topographical variations emerged. This behavior reflects a selective surface reorganization driven by localized surface erosion dynamics, in which the uppermost asperities undergo preferential dissolution as a consequence of acid-induced polymer chain scission and hydrolytic degradation at the interface [28,29]. Importantly, no perforation or macroscopic degradation compromising the integrity of the capsule was observed, confirming that the polymeric shell retained its chemical and structural stability under simulated gastric conditions even after 4 h of acidic exposure, well beyond the typical transit times in the gastric tract (~1–2 h).

Concurrently, contact angle measurements showed a marked increase in hydrophilicity, with the water contact angle decreasing from 79.2° to 62.2° after acid treatment (Figure 5b). The increased hydrophilicity can be attributed to the protonation and partial hydrolysis of ester bonds in polylactic acid (PLA) under acidic conditions. This process leads to the formation of hydroxyl (-OH) and carboxylic (-COOH) terminal groups on the surface, enhancing its polarity and affinity for water molecules [30,31].

### 3.4. Mechanical Characterization of Hollow Capsule

**Figure 6:**
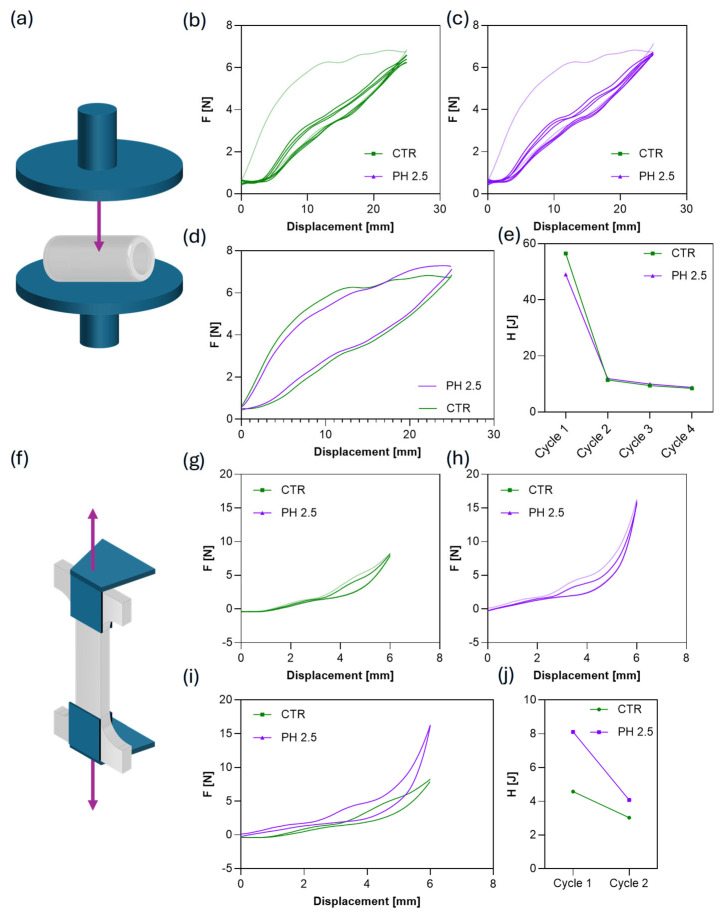
(**a**) Schematic illustration of the compression setup. Purple arrow indicates force vector. Force-displacement curves obtained cyclically compressing hollow capsules after 2 h of immersion in PBS at (**b**) pH 7.4 and (**c**) pH 2.5. (**d**) Force-displacement curves after the first compression cycle. (**e**) Capsule hysteresis as a function of the compression cycle. (**f**) Schematic illustration of the tensile setup. Purple arrows indicate force vectors. Force-displacement curves obtained from cyclically stretching dog-bone-shaped BioFlex specimens after 2 h of immersion in PBS at (**g**) pH 7.4 and (**h**) pH 2.5. (**i**) Force-displacement curves after the first stretching cycle. (**j**) Specimen hysteresis as a function of the stretching cycle.

The printed polymer was tested under different loading configurations to estimate its mechanical response during the simulated delivery process. Specifically, control (CTR) and acid-treated capsules were subjected to compression along their minor axis (Figure 6a) to assess their ability to withstand mechanical stresses typical of gastrointestinal transit without structural failure or deformation that could compromise the release profile. In addition, 3D-printed dog-bone specimens (Figure 6f) were monitored under cyclic tensile testing to evaluate potential changes in hysteresis behavior upon repeated deformation. The compression tests shown in Figure 6b–d reveal similar force–displacement profiles for CTR (pH 7.4) and acid-treated (pH 2.5) samples, with overlapping trends in the elastic region and at maximum load. This indicates that the short-term acidic treatment did not significantly affect the bulk mechanical stiffness or load-bearing capacity of the printed shell. Figure 6g–i shows the tensile cycles of both the control and pH 2.5 groups. The force–displacement curves exhibit a comparable nonlinear trend, with a gradual increase in load during extension and partial recovery upon unloading. The first loading cycle shows a markedly higher hysteresis area compared to subsequent ones, indicating a viscoelastic relaxation of the polymeric network typical of thermoplastic materials under cyclic strain. The hysteresis (H) in Figure 6e,j confirms this behavior, showing a sharp decrease from cycle 1 to cycle 2 and stabilization in the following cycles for both CTR and acidic conditions in compression and tensile tests.

### 3.5. Oil Encapsulation with and Without Xanthan Gum

**Figure 7:**
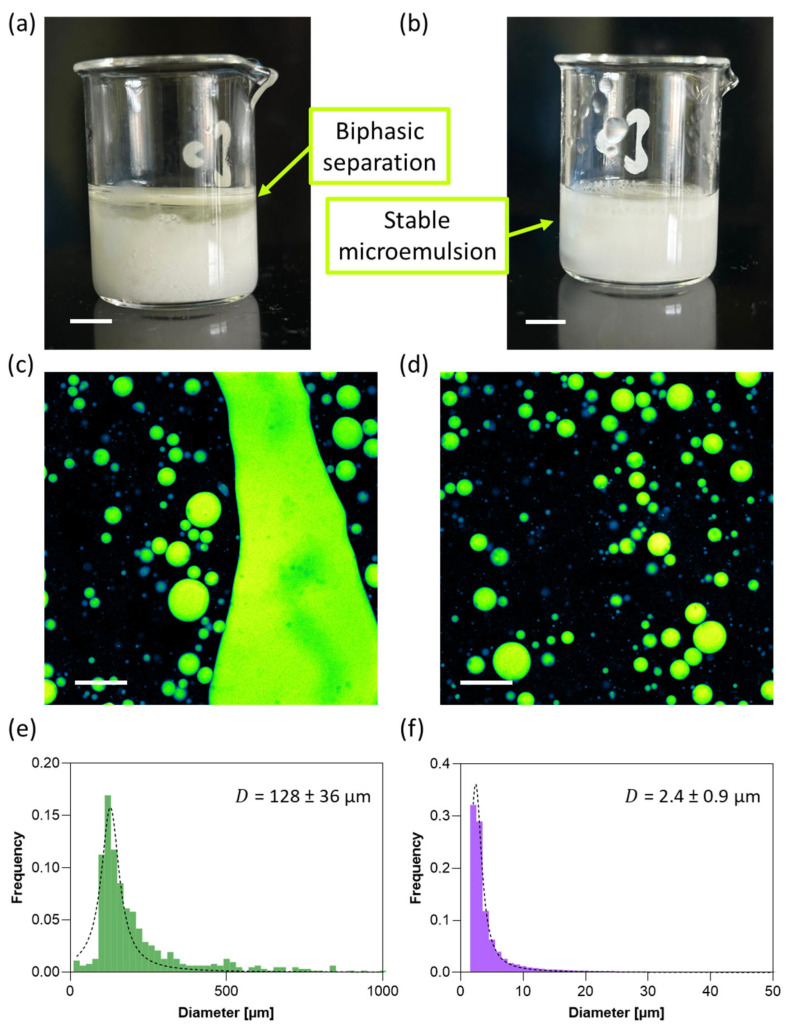
Representative pictures of alginate/essential oil blend (**a**) without and (**b**) with xanthan gum. Scale bar: 5 mm. (**c**,**d**) Confocal fluorescence images of autofluorescent oil droplets and (**e**,**f**) corresponding diameter distributions in alginate/essential oil and alginate/xanthan gum/essential oil blends, respectively. Scale bar: 100 µm.

Achieving a stable microemulsion is crucial to ensure long-term homogeneity, confinement of the encapsulated phase, and controlled release of hydrophobic actives. In oil-based delivery systems, instability phenomena such as coalescence, creaming, or phase separation can severely compromise both dosage precision and release kinetics. To overcome these limitations, we formulated a hydrogel blend composed of sodium alginate and xanthan gum, which provides complementary structural and rheological properties. Alginate offers biocompatibility and mild gelation under physiological conditions, forming a hydrated matrix that can encapsulate and immobilize oil droplets. However, alginate alone often exhibits limited viscosity and mechanical stability, which may lead to droplet migration or fusion over time (Figure 7a). The addition of xanthan gum, an anionic polysaccharide with high molecular weight and shear-thinning behavior, enhances the viscoelasticity of the matrix and promotes more uniform dispersion of oil droplets. Moreover, xanthan gum increases the water-binding capacity of the blend, reducing syneresis and preventing phase separation during storage (Figure 7b).

Fluorescence confocal microscopy revealed clear differences in the distribution and morphology of autofluorescent oil droplets depending on the formulation. In the absence of xanthan gum (Figure 7c), the fluorescent signal indicated a broad and heterogeneous droplet size distribution, with an average diameter of 128 µm (Figure 7e). In contrast, the addition of xanthan gum resulted in a markedly narrower and more uniform distribution (Figure 7d), with a mean droplet diameter of 2.4 µm (Figure 7f). This enhanced uniformity confirms that xanthan gum improves emulsion stability by increasing the viscosity of the hydrogel matrix and minimizing droplet coalescence during mixing and gel formation.

### 3.6. Drug Release Profile

Designing delivery systems capable of precisely regulating the release of multiple bioactive compounds remains a central goal in enteric drug delivery. Beyond mere encapsulation, an effective carrier must ensure temporal coordination between distinct drugs, protect them during gastrointestinal transit, and release them at the appropriate intestinal site. A dual-compartment capsule architecture housing different hydrogel blends offers a smart strategy to achieve such control, allowing independent modulation of release kinetics.

**Figure 8:**
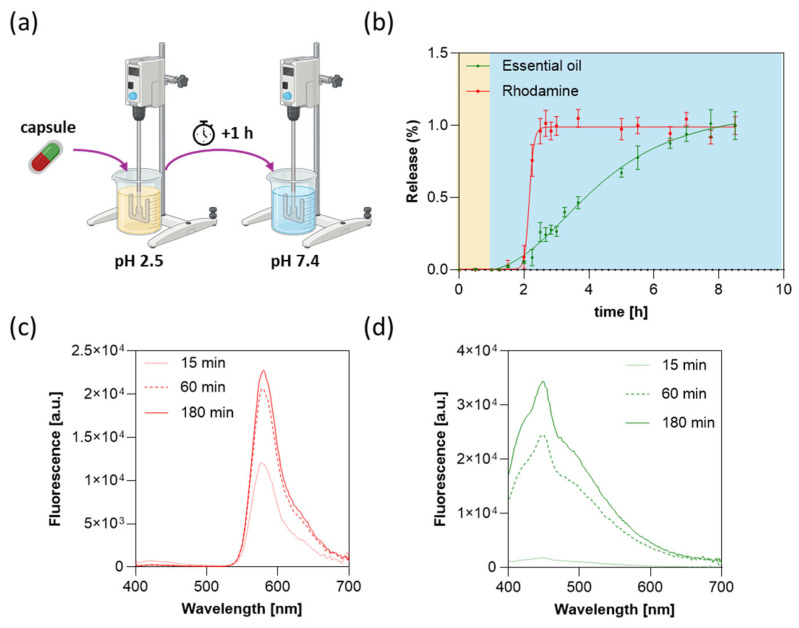
(**a**) Schematic illustration of the release experiment. (**b**) Percentage of rhodamine B (red) and rosemary essential oil (green) release over time. Capsules were kept at pH 2.5 for 1 h (yellow region) and then transferred to pH 7.4 (light blue region). Data are represented as mean value ± standard deviation of three independent experiments. Fluorescence spectra of (**c**) rhodamine B and (**d**) rosemary essential oil as a function of release time in PBS at pH 7.4.

The capsule outlined in Figure 1 was designed to pass intact through the stomach thanks to the Eudragit film while releasing gelatin- and alginate-based blends sequentially in the small intestine. Hydrogel blends and gastroprotective film were extruded in a single printing session, allowing high-throughput fabrication of a large-scale batch of printed capsules with minimal intra-batch variability. Capsules were first incubated in a simulated gastric fluid (pH 2.5) to assess leakage resistance under acidic conditions (Figure 8a). After 1 h, no detectable fluorescence was observed, confirming the structural integrity of the capsule and complete retention of the encapsulated material in the gastric environment (Figure 8b). Upon transfer to a neutral medium at pH 7.4 (Figure 8a), we observed a 1 h delay due to the Eudragit film dissolution at a rate of 5 µm/min, followed by a sharp increase in rhodamine fluorescence, indicative of a rapid dissolution of the outermost gelatin compartment once the pH-sensitive cap was removed (Figure 8b,c).

The release of the essential oil was monitored through oil autofluorescence, which exhibited a slower, more gradual increase with an average release rate of 8.3 µL/h (Figure 8b,d). This kinetic behavior arises from two synergistic factors: (i) the oil resides in the innermost compartment, providing additional protection from external perturbations; and (ii) the viscoelastic nature of the alginate–xanthan gum matrix retards both diffusion and matrix dissolution, leading to a sustained and controlled release [32].

### 3.7. Capsule Stability and Inter-Compartmental Cross-Contamination

**Figure 9:**
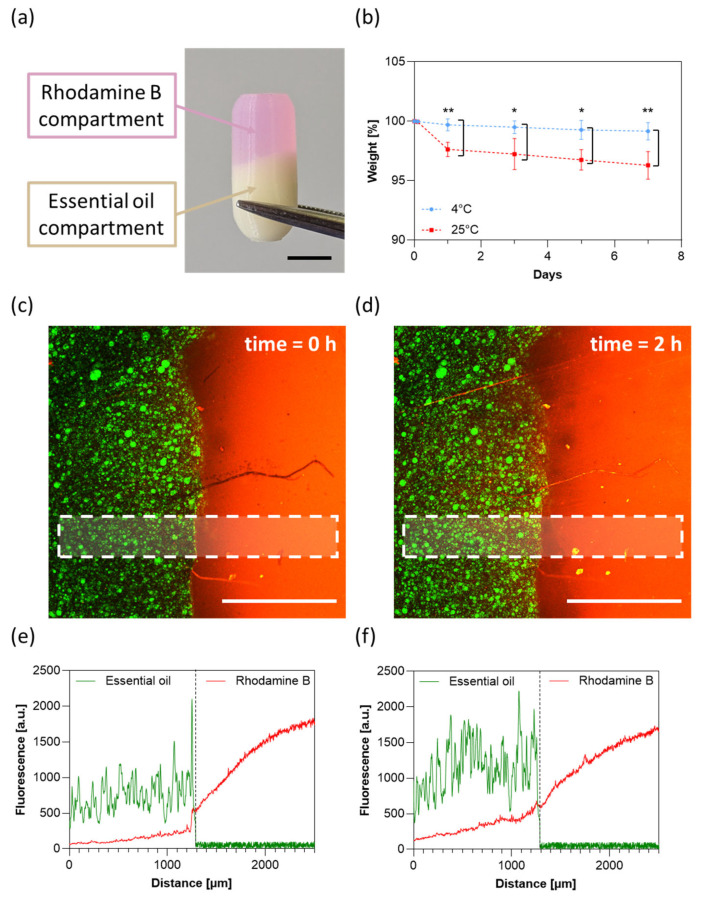
(**a**) Representative picture of the 3D printed capsule. Scale bar: 5 mm. (**b**) Weight profiles of capsules under different temperature conditions. Data are represented as mean value ± standard deviation of three independent experiments. Statistical significance was evaluated using the two-way ANOVA test (* *p* < 0.05; ** *p* < 0.01). Confocal fluorescence images of the interface between rosemary essential oil (green) and rhodamine B (red) compartments at (**c**) 0 h and (**d**) 2 h. Scale bar: 1 mm. Corresponding fluorescence intensities at (**e**) 0 h and (**f**) 2 h were evaluated longitudinally within the white dashed area highlighted in panels (**c**) and (**d**), respectively.

Figure 9a shows the final prototype of the capsule, in which rhodamine compartment and essential oil compartment are clearly dinstinct. The stability of the capsules was evaluated by monitoring their weight over 7 days (Figure 9b). Capsules stored at 4 °C and 50% RH demonstrated excellent stability, exhibiting negligible mass change throughout the experiment. In contrast, capsules stored at 25 °C and 50% RH showed a progressive reduction in weight up to 4% after 7 days, indicating that lower storage temperatures are critical for preserving the capsule integrity.

To assess the compartmentalization efficiency without mechanically compromising the capsule structure, we replicated the printing protocol using a squared capsule with a removable face and examined potential intermixing between the essential oil compartment and the rhodamine B compartment. As shown in Figure 9c, the two regions remained sharply delineated immediately after printing, with clear boundaries separating the rosemary essential oil (left) from the rhodamine B (right), confirming that the printing process establishes distinct reservoirs with negligible cross-contamination (Figure 9e). After 2 h of incubation, a slight diffusion of rhodamine B into the adjacent compartment was observed (Figure 9d,f). This slow migration is consistent with the small molecular size and high diffusivity of rhodamine B in hydrated hydrogel matrices. Importantly, such diffusion is expected to be substantially reduced for larger molecules or probiotics, indicating that the printing strategy provides sufficient compartmental segregation.

## 4. Conclusions

This work demonstrates the feasibility of using a one-step 3D printing strategy to fabricate multicompartment capsules capable of achieving spatially controlled and tunable release profiles. By integrating polymeric materials with distinct physicochemical and pH-responsive properties, we achieved a design that allows sequential release of both hydrophilic and hydrophobic loads within physiologically relevant environments. Such modularity represents an important step toward the next generation of intelligent drug delivery systems and bioactive carriers.

Nevertheless, several challenges remain before full clinical translation can be realized. The development of optimized bioinks that simultaneously ensure printability, biocompatibility, and mechanical stability remains a central challenge. Furthermore, the single-step fabrication approach, while advantageous in reducing post-processing, demands tight control over polymer rheology, crosslinking kinetics, and interfacial adhesion between compartments. Issues related to scalability, long-term storage stability, and regulatory compliance will also need to be systematically addressed to enable reproducible manufacturing and clinical-grade validation.

Despite these hurdles, the demonstrated ability to design and print structurally complex and functionally compartmentalized capsules in a reproducible and programmable manner represents a substantial advancement over conventional microencapsulation and coating-based techniques. The versatility of this platform extends well beyond the conceptual model used here, offering significant potential for personalized medicine. Specifically, this technology paves the way for the development of personalized oral chemotherapy plans, where patient-specific dosing and targeted release are critical to reducing systemic toxicity [33]. Additionally, the precise spatial control over release kinetics opens new therapeutic opportunities for the management of chronic conditions such as inflammatory bowel diseases (IBD), enabling the localized delivery of anti-inflammatory agents directly to affected intestinal segments [33].

## Data Availability

Data available from the corresponding authors upon reasonable request.

## References

[1] Bácskay I., Ujhelyi Z., Fehér P., Arany P. (2022). The Evolution of the 3D-Printed Drug Delivery Systems: A Review. Pharmaceutics.

[2] Maroni A., Melocchi A., Parietti F., Foppoli A., Zema L., Gazzaniga A. (2017). 3D printed multi-compartment capsular devices for two-pulse oral drug delivery. J. Control. Release.

[3] Kapoor D.U., Pareek A., Uniyal P., Prajapati B.G., Thanawuth K., Sriamornsak P. (2025). Innovative applications of 3D printing in personalized medicine and complex drug delivery systems. iScience.

[4] Choudhury D., Murty U.S., Banerjee S. (2021). 3D printing and enteric coating of a hollow capsular device with controlled drug release characteristics prepared using extruded Eudragit^®^ filaments. Pharm. Dev. Technol..

[5] Chansatidkosol S., Limmatvapirat C., Sriamornsak P., Akkaramongkolporn P., Thanawuth K., Krongrawa W., Limmatvapirat S. (2025). 3D-Printed Shellac-Based Delivery Systems: A Biopolymer Platform for Intestinal Targeting of Biologically Active Compounds. ACS Omega.

[6] Muehlenfeld C., Duffy P., Yang F., Pérez D.Z., El-Saleh F., Durig T. (2024). Excipients in Pharmaceutical Additive Manufacturing: A Comprehensive Exploration of Polymeric Material Selection for Enhanced 3D Printing. Pharmaceutics.

[7] Alzhrani R.F., Fitaihi R.A., Majrashi M.A., Zhang Y., Maniruzzaman M. (2025). Toward a harmonized regulatory framework for 3D-printed pharmaceutical products: The role of critical feedstock materials and process parameters. Drug Deliv. Transl. Res..

[8] Shojaie F., Ferrero C., Caraballo I. (2023). Development of 3D-Printed Bicompartmental Devices by Dual-Nozzle Fused Deposition Modeling (FDM) for Colon-Specific Drug Delivery. Pharmaceutics.

[9] Wang S., Chen X., Han X., Hong X., Li X., Zhang H., Li M., Wang Z., Zheng A. (2023). A Review of 3D Printing Technology in Pharmaceutics: Technology and Applications, Now and Future. Pharmaceutics.

[10] Saleh-Bey-Kinj Z., Heller Y., Socratous G., Christodoulou P. (2025). 3D Printing in Oral Drug Delivery: Technologies, Clinical Applications and Future Perspectives in Precision Medicine. Pharmaceuticals.

[11] Park B.J., Choi H.J., Moon S.J., Kim S.J., Bajracharya R., Min J.Y., Han H.-K. (2018). Pharmaceutical applications of 3D printing technology: Current understanding and future perspectives. J. Pharm. Investig..

[12] Hu J., Wan J., Xi J., Shi W., Qian H. (2024). AI-driven design of customized 3D-printed multi-layer capsules with controlled drug release profiles for personalized medicine. Int. J. Pharm..

[13] Xu P., Nguyen H.T., Huang S., Tran H. (2024). Development of 3D-Printed Two-Compartment Capsular Devices for Pulsatile Release of Peptide and Permeation Enhancer. Pharm. Res..

[14] Algahtani M.S., Ahmad J., Mohammed A.A., Ahmad M.Z. (2024). Extrusion-based 3D printing for development of complex capsular systems for advanced drug delivery. Int. J. Pharm..

[15] Asadi M., Salehi Z., Akrami M., Hosseinpour M., Jockenhövel S., Ghazanfari S. (2023). 3D printed pH-responsive tablets containing N-acetylglucosamine-loaded methylcellulose hydrogel for colon drug delivery applications. Int. J. Pharm..

[16] Millet E., O’sHea J.P., Griffin B.T., Dumont C., Jannin V. (2025). Next generation capsules: Emerging technologies in capsule fabrication and targeted oral drug delivery. Eur. J. Pharm. Sci..

[17] Salawi A. (2022). Pharmaceutical Coating and Its Different Approaches, a Review. Polymers.

[18] Kantaros A., Ganetsos T., Petrescu F.I.T., Ungureanu L.M., Munteanu I.S. (2024). Post-Production Finishing Processes Utilized in 3D Printing Technologies. Processes.

[19] Charbe N.B., McCarron P.A., Lane M.E., Tambuwala M.M. (2017). Application of three-dimensional printing for colon targeted drug delivery systems. Int. J. Pharm. Investig..

[20] Melocchi A., Parietti F., Maccagnan S., Ortenzi M.A., Antenucci S., Briatico-Vangosa F., Maroni A., Gazzaniga A., Zema L. (2018). Industrial Development of a 3D-Printed Nutraceutical Delivery Platform in the Form of a Multicompartment HPC Capsule. Aaps Pharmscitech.

[21] Xie Y., Zhang K., Zhu J., Ma L., Zou L., Liu W. (2024). Shell–Core Microbeads Loaded with Probiotics: Influence of Lipid Melting Point on Probiotic Activity. Foods.

[22] Wendel U. (2022). Assessing Viability and Stress Tolerance of Probiotics—A Review. Front. Microbiol..

[23] Walczak-Skierska J., Ludwiczak A., Sibińska E., Pomastowski P. (2024). Environmental Influence on Bacterial Lipid Composition: Insights from Pathogenic and Probiotic Strains. ACS Omega.

[24] Haryńska A., Carayon I., Kosmela P., Szeliski K., Łapiński M., Pokrywczyńska M., Kucińska-Lipka J., Janik H. (2020). A comprehensive evaluation of flexible FDM/FFF 3D printing filament as a potential material in medical application. Eur. Polym. J..

[25] U.S. Department of Health and Human Services, Food and Drug Administration (2015). Size, Shape, and Other Physical Attributes of Generic Tablets and Capsules Guidance for Industry.

[26] European Union (2013). EMA/CHMP/QWP/805880/2012 Rev. 2, Guideline on pharmaceutical development of medicines for paediatric use, European Medicines Agency, Committee for Medicinal Products for Human Use.

[27] Pitt C.G., Hendren R., Schindler A., Woodward S.C. (1984). The enzymatic surface erosion of aliphatic polyesters. J. Control. Release.

[28] Vaid R., Yildirim E., Pasquinelli M.A., King M.W. (2021). Hydrolytic Degradation of Polylactic Acid Fibers as a Function of pH and Exposure Time. Molecules.

[29] Rowe M.D., Eyiler E., Walters K.B. (2016). Hydrolytic degradation of bio-based polyesters: Effect of pH and time. Polym. Test..

[30] Woodard L.N., Grunlan M.A. (2018). Hydrolytic Degradation and Erosion of Polyester Biomaterials. ACS Macro Lett..

[31] Minopoli A., Evangelista D., Marras M., Perini G., Palmieri V., De Spirito M., Papi M. (2025). Viscoelastic interpretation of AFM nanoindentation for predicting nanoscale stiffness in soft biomaterials. Polym. Test..

[32] Ducray F., Ramirez C., Robert M., Fontanilles M., Bronnimann C., Chinot O., Estrade F., Durando X., Cartalat S., Bastid J. (2023). A Multicenter Randomized Bioequivalence Study of a Novel Ready-to-Use Temozolomide Oral Suspension vs. Temozolomide Capsules. Pharmaceutics.

[33] Munoz-Perez E., Santos-Vizcaino E., Goyanes A., Basit A.W., Hernandez R.M. (2025). 3D-Printed core-shell tablet for effective oral delivery of AT-MSC secretome in inflammatory bowel disease therapy. Drug Deliv. Transl. Res..

